# Structural and Functional Studies of a Newly Grouped *Haloquadratum walsbyi* Bacteriorhodopsin Reveal the Acid-resistant Light-driven Proton Pumping Activity*

**DOI:** 10.1074/jbc.M115.685065

**Published:** 2015-10-19

**Authors:** Min-Feng Hsu, Hsu-Yuan Fu, Chun-Jie Cai, Hsiu-Pin Yi, Chii-Shen Yang, Andrew H.-J. Wang

**Affiliations:** From the ‡ Institute of Biological Chemistry and; § Core Facilities for Protein Structural Analysis, Academia Sinica, Taipei 11529 and; the ¶ Department of Biochemical Science and Technology, College of Life Science,; ‖ Yen Tjing Ling Industrial Research Institute, and; ** Institute of Biotechnology, College of Bio-Resources and Agriculture, National Taiwan University, Taipei 10617, Taiwan

**Keywords:** crystal structure, membrane protein, proton pump, rhodopsin, site-directed mutagenesis, bacteriorhodopsin, lipid cubic phase, spectral-tuning, structure

## Abstract

Retinal bound light-driven proton pumps are widespread in eukaryotic and prokaryotic organisms. Among these pumps, bacteriorhodopsin (BR) proteins cooperate with ATP synthase to convert captured solar energy into a biologically consumable form, ATP. In an acidic environment or when pumped-out protons accumulate in the extracellular region, the maximum absorbance of BR proteins shifts markedly to the longer wavelengths. These conditions affect the light-driven proton pumping functional exertion as well. In this study, wild-type crystal structure of a BR with optical stability under wide pH range from a square halophilic archaeon, *Haloquadratum walsbyi* (*Hw*BR), was solved in two crystal forms. One crystal form, refined to 1.85 Å resolution, contains a trimer in the asymmetric unit, whereas another contains an antiparallel dimer was refined at 2.58 Å. *Hw*BR could not be classified into any existing subgroup of archaeal BR proteins based on the protein sequence phylogenetic tree, and it showed unique absorption spectral stability when exposed to low pH values. All structures showed a unique hydrogen-bonding network between Arg^82^ and Thr^201^, linking the BC and FG loops to shield the retinal-binding pocket in the interior from the extracellular environment. This result was supported by R82E mutation that attenuated the optical stability. The negatively charged cytoplasmic side and the Arg^82^–Thr^201^ hydrogen bond may play an important role in the proton translocation trend in *Hw*BR under acidic conditions. Our findings have unveiled a strategy adopted by BR proteins to solidify their defenses against unfavorable environments and maintain their optical properties associated with proton pumping.

## Teaser

**Background:** Most bacteriorhodopsins demonstrate red-shifted spectrum in acidic condition.

**Results:** Structures of *Haloquadratum walsbyi* bacteriorhodopsin explain stable action spectra from pH 2 to 8.

**Conclusion:** The extracellular hydrogen-bonding network assists in the maintenance of protonation status in the *Haloquadratum walsbyi* bacteriorhodopsin retinal-binding pocket.

**Significance:** A bacteriorhodopsin subfamily has a stable optical property, and its structure is useful for protein engineering in optogenetic tools.

## Introduction

Retinal bound integral membrane proteins, rhodopsins, in diverse species of life utilize solar energy for various functions such as ion translocation, photosensing, and channel activity (1, 2). Several types of microbial rhodopsins that are proton pumps have been found in diverse microorganisms: bacteriorhodopsin (BR),^5^ blue proteorhodopsin (BPR), green proteorhodopsin (GPR), actinorhodopsin (ActR), *Volvox carteri* rhodopsin (VChR), *Exiguobacterium sibiricum* rhodopsin (ESR), and *Salinibacter ruber* xanthorhodopsin (XR) (3–8). Recently, some of these rhodopsins have been applied as important tools for optogenetic control of cells, tissue, and animals (9).

The light-driven proton pumps feature a seven-transmembrane α-helical region with a lysine-bound retinal that serves as a chromophore responsive to light. These BR proteins respond to ∼550 nm light and exert outward proton pumping, resulting in a proton gradient in the extracellular region (10–13). These proteins consequently facilitate the inflow of protons back into the cell through ATP synthase to generate ATP (14). The first and most well studied BR from *Halobacterium salinarum*, *Hs*BR (10), was shown to be optically and functionally durable under heat and high salinity conditions (15), making it one of the most stable membrane proteins. However, BR proteins have a well established and significant property, a red-shifted activity spectrum at acidic pH, wherein a maximum red-shift of ∼55 nm in λ_max_ for *Hs*BR is from protonation of an aspartate residue at the retinal Schiff base (Asp^85^ in *Hs*BR) (16–18). At acidic pH, the lack of proton transport is due to the protonated aspartate, which should be the proton acceptor for the Schiff base during the photocycle; in the absence of an acceptor, the proton transfer cannot take place, and a critical step in the transport does not occur. Those results are expected because releasing a proton from a protein into an environment of low pH is not chemically favored, and a red-shifted action spectrum is a conventional indicator for the protonated aspartate in the Schiff base binding pocket.

Most BR proteins are not functional under acidic conditions. After we reported a BR from *Haloarcula marismortui*, *Hm*BRII, BR proteins started to surface in the past few years. *Hm*BRII showed high optical stability in acidic conditions even down to pH of 1.6 and maintained its light-driven proton pumping activity at pH of 4.0 (19). This observation was extended when we identified another BR from *Haloquadratum walsbyi*, *Hw*BR, which showed optical durability in acidic conditions.^6^

After the structures of *Hs*BR were solved by electron microscopy in 1996 (20) and by x-ray in 1997 (21), more than 70 *Hs*BR structures of different length proteins, intermediates, mutants, and binding statuses have been reported, and the molecular mechanism of light-driven proton transportation was described in detail (11, 13, 22). Furthermore, six BR-like crystal structures were determined in the last decade, including bacteriorhodopsin (bR) from *H. salinarum* (21, 23, 24), archaerhodopsin-1 and -2 (aR-1 and aR-2) from *Halorubrum* sp. aus-1 and -2 (25, 26), deltarhodopsin-3 (dR3) from *Haloterrigena thermotolerans* (27), and cruxrhodopsin-3 (cR-3) from *Haloarcula vallismortis* (28). Moreover, structural information also became available for seven light-driven proton translocators identified from bacteria and eukaryota, xanthorhodopsin (XR) from *S. ruber* (29), *Acetabularia* rhodopsin (ARII) from the marine plant *Acetabularia acetabulum* (30), channelrhodopsin (ChR) chimera from *Chlamydomonas reinhardtii* (31), and both blue and green proteorhodopsin (BPR and GPR) (32–34). However, none of them has a relatively consistent activity spectrum in broad pH conditions.

Here, we report the atomic structure, sequence analysis, and photochemical properties of a BR protein, *Hw*BR, and we propose that *Hw*BR belongs to a new subfamily of BRs that we have named qR. The crystal structures of *Hw*BR revealed that a unique arginine residue stabilizes the extracellular loop region by forming hydrogen bonds with a threonine residue located in the membrane edge of extracellular region. The importance of this local structure, which shields the interior environment of *Hw*BR from the low pH extracellular area, was further validated by the mutagenesis approach.

## Experimental Procedures

### 

#### 

##### Phylogenomic Analysis

Thirteen BR-like amino acid sequences were used for phylogenomic analysis^7^. The unweighted pair group method with arithmetic mean (UPGMA) algorithm was employed in this work, with the Kimura protein distance measure from CLC sequence Viewer 6.9. The bootstrap is based on 100 replicates. The abbreviations are as follows: Hr.sodomense_aR3 (archaerhodopsin-3 in *Halorubrum sodomense*, National Center for Biotechnology Information (NCBI) GenBank^TM^ BAA09452.1), Hr.sp.aus-1_aR1_1UAZ (archaerhodopsin-1 in *Halorubrum* sp. *aus-1* or also known as *Halorubrum chaoviator*, Protein Data Bank (PDB) ID 1UAZ), Hr.sp.aus-2_aR2_1VGO (archaerhodopsin-2 in *Halorubrum* sp. *aus-2*, PDB ID 1VGO), Hq.walsbyi_BR (bacteriorhodopsin in *H. walsbyi*, NCBI Gene ID 4193772), Ha.marismortui_BRII (*Hm*BRII in *H. marismortui*, NCBI Gene ID 3128157), Hb.salinarum_bR_1C3W (bacteriorhodopsin in *H. salinarum*, PDB ID 1C3W), Ha.japonica_cR (cruxrhodopsin in *Haloarcula japonica*, NCBI GenBank BAA81816.1), Ha.argentinensis_cR1 (cruxrhodopsin-1 in *Haloarcula argentinensis*, NCBI Gene ID 1060883), Ha.vallismortis_cR3 (cruxrhodopsin-3 in *H. vallismortis*, NCBI Gene ID 1769808), Ha.marismortui_BRI (*Hm*BRI in *H. marismortui*, NCBI Gene ID 3128463), Ha.hispanica_BR (bacteriorhodopsin in *Haloarcula hispanica*, NCBI Gene ID 11049305), Ha.sp.arg-2_cR2 (cruxrhodopsin-2 in *Haloarcula* sp. *Arg-2*, NCBI Gene ID 2499387), and Ha.thermotolerans_dR3 (deltarhodopsin-3 in *H. thermotolerans*, PDB ID 4FBZ). Sequence information for the other rhodopsins was obtained from GenBank and the Kyoto Encyclopedia of Genes and Genomes (KEGG) database.

##### Bacterial Strains and Expression of BR from H. walsbyi

Routine DNA manipulations were carried out according to standard molecular cloning procedures. The *Hw*BR gene was cloned into a pUC57 vector using directed synthesis. The final sequence was CC^1^ATGgCT*XXXX*^753^GACCTCGAG (underlining indicates the restriction sites for NcoI and XhoI, respectively, and *g* indicates a modified base.) The DNA fragments were obtained by NcoI and XhoI and were then inserted into the NcoI and XhoI sites of the pET-21d vector (Novagen). Consequently, a plasmid encoding hexahistidines at the C terminus was constructed. *Hw*BR gene mutants were generated using the QuikChange site-directed mutagenesis method (Stratagene). The constructed plasmids were confirmed to have the expected nucleotide sequence using an automated sequencer.

##### Protein Expression in Escherichia coli and Purification

*Hw*BR protein with hexahistidines at the C terminus was expressed in *E. coli* C43(DE3). The protein was purified using nickel-nitrilotriacetic acid resin chromatography (GE Healthcare) as described previously (35) and was solubilized in 0.05% *n*-dodecyl-β-d-maltoside (DDM).

##### UV-visible Spectroscopy

The purified sample was concentrated and exchanged to a buffer containing 4 m NaCl, 50 mm Tris-HCl, and 0.05% DDM using an Amicon apparatus (Millipore). UV-visible spectra were recorded using a U-1900 spectrophotometer (Hitachi). pH-dependent spectra and a titration curve were conducted as described in previous research (36). The temperature was maintained at 298 K.

##### Photocurrent Measurement

The electrochemical cell was designed by Chu *et al.* (37) and modified in our previous work (19). Photocurrent measurements of purified proteins were carried out by using a modulated continuous wave (CW) 532-nm green laser as the excitation light source and controlled by a data acquisition card.

##### Light-driven Proton Transport Activity

Light-driven proton transport activity was measured by monitoring light-induced pH changes using a glass electrode in real time. *E. coli* cells expressing the target rhodopsin were harvested by centrifugation (4,800 × *g* for 10 min). They were then washed three times and resuspended in measurement buffer (10 mm NaCl, 10 mm MgSO_4_, and 100 μm CaCl_2_). The concentration of the cell suspension was adjusted to obtain an *A*_600_ ∼2.0; the suspension was maintained in the dark and then illuminated with a green CW laser at 1 watt (532 nm). A parallel experiment with 10 μm carbonyl cyanide *m*-chlorophenyl hydrazone (CCCP) was conducted to confirm the proton-specificity of the assay.

##### Protein Preparation for Crystallography

To screen for the optimal *Hw*BR crystallization conditions, purified *Hw*BR was analyzed for monodispersity using a size exclusion column, and its absorption was monitored at 280 and 552 nm. The protein was loaded onto a size exclusion column (Superdex 200 10/30 GL; GE Health Sciences) using Buffer A (50 mm CH_3_COONa, pH 4.5, 200 mm NaCl) and Buffer B (20 mm Tris, pH 7.0, 150 mm NaCl) as the elution buffer in the presence of 0.05–0.15% DDM, *n*-octyl-β-d-glucopyranoside (OG), or *n*-decyl-β-maltoside (DM). After dialysis in buffer with 0.15% DM, the elution pattern showed a monodispersed peak as well. In our detergent screening experiment, the *Hw*BR protein showed monodisperse peaks in both buffers in 0.15% DM. Therefore, we used *Hw*BR protein in Buffer A with 0.15% DM as the sample for crystallization.

##### Crystallization and X-ray Diffraction Data Collection

The purified *Hw*BR protein was concentrated to ∼17 mg/ml, as estimated by ultraviolet absorbance, and it was mixed with 1-oleoyl-rac-glycerol (monoolein; Sigma-Aldrich) at a 2:3 (w/w) protein-to-lipid ratio using the twin-syringe mixing method. The volume of each drop was 0.2 μl of protein-lipid mixture plus 1 μl. *Hw*BR crystals of the trimeric form were grown in 0.05 m sodium citrate, pH 5.5, 0.05 m NaCl, and 15% (v/v) PEG 400, and antiparallel dimeric crystals were grown in 0.1 m ammonium sulfate, 0.1 m sodium chloride, 0.01 m sodium acetate, pH 4.0, and 16.5% (v/v) PEG 200. The size of the crystals reached about 50 × 50 × 5 μm within 2–30 days at 20 °C. The *Hw*BR trimeric form crystals were soaked in 30% (v/v) glycerol as a cryoprotectant before harvest.

X-ray diffraction data were collected at BL15A1 of the National Synchrotron Radiation Research Center (NSRRC), Hsinchu, Taiwan and at 44XU of SPring-8, Sayo, Japan. The data were processed using HKL2000 (53). We obtained the phases by molecular replacement using archaerhodopsin-2 as a template (1VGO) (25). The PHENIX (38), refmac5 (39), and COOT (40) programs were used for molecular replacement, structural refinement, and structural viewing, respectively. All structure figures were prepared in PyMOL (Schrödinger, LLC).

## Results

### 

#### 

##### Sequence Analysis of BR-like Proteins

**FIGURE 1.**
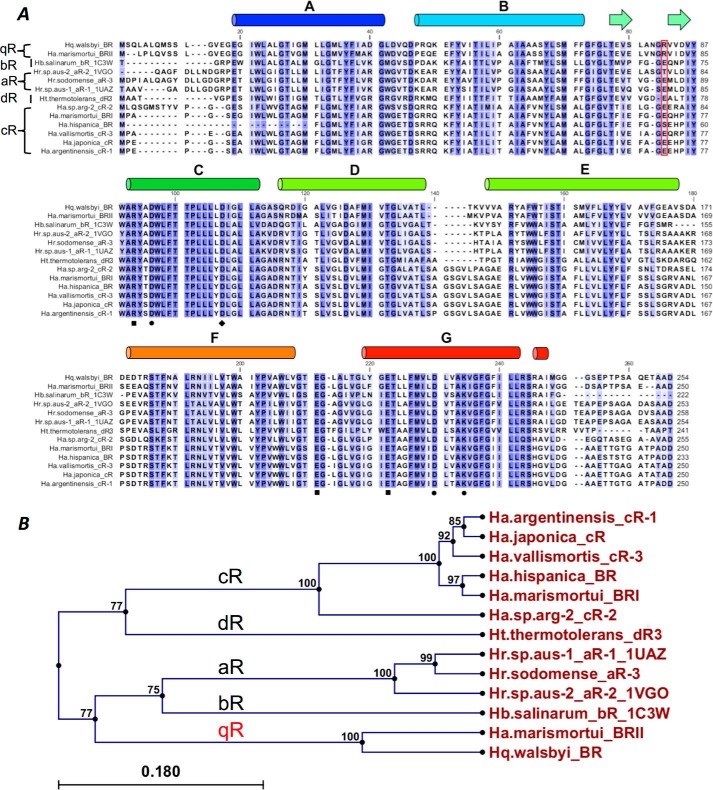
**Sequence alignment of the light-driven proton pumps in halobacteria.** *A*, 13 amino acid sequences of BR-like proteins were aligned. The key residues are annotated with different symbols. *Circle*, retinal-binding pocket; *diamond*, proton reuptake residue; *square*, proton releasing group. The key residue, Arg^82^ (*Hw*BR), in this study is marked by the *red box*. The secondary-structural information of *Hw*BR is shown above the alignment. *B*, phylogenomics analysis of the amino acid sequences of the light-driven proton pumps in halobacteria. The analysis classified *Hw*BR from a quadrate-shaped bacteria into a new separate superfamily, qR.

A previous study (41) compared the BR protein sequences of several species living in different environments, including salterns (42), spring areas (43), and others (44–46), and reported BR subgroups designated aR to dR (47). Multiple alignment of BR amino acid sequences (Fig. 1*A*) shows that the BRs share 50–80% identity. However, the amino acid sequence of *Hw*BR from a quadrate-shaped bacterium, *H. walsbyi*, constitutes a novel group with *Hm*BRII according to the phylogenetic tree analysis (Fig. 1*B*). We named this unidentified and distinct superfamily qR.

##### Purification of Monodispersed HwBR Proteins

**FIGURE 2.**
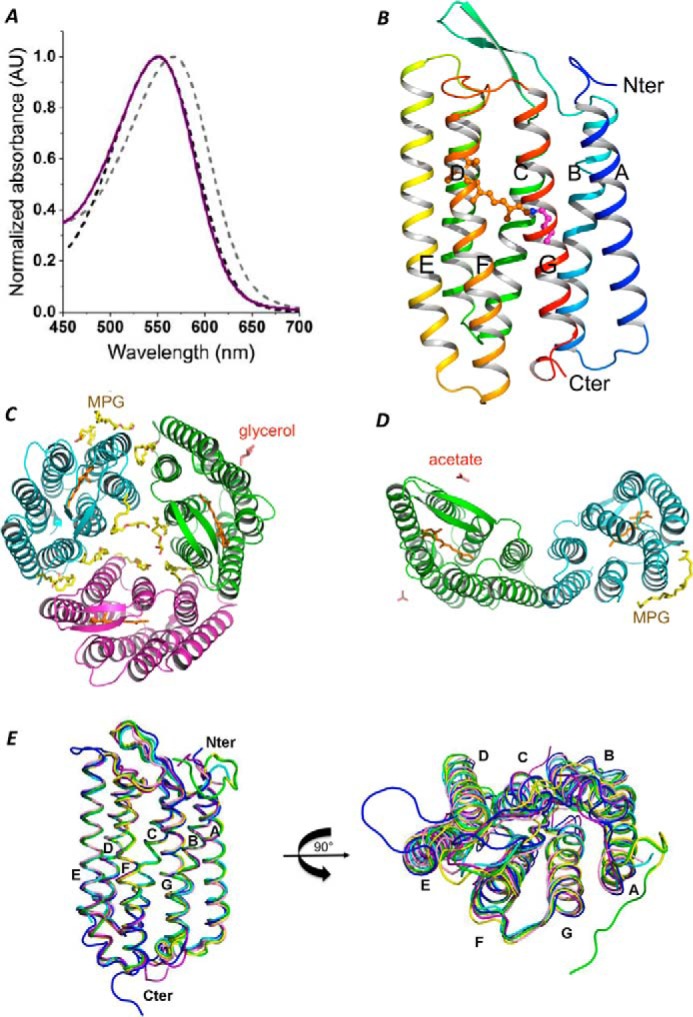
**Optical property and overall structure of wild-type and D93N *Hw*BR proteins.** *A*, the UV-visible spectra of purple membrane (trimeric *Hs*BR) (*gray dashed line*), monomeric *Hs*BR (*black dashed line*), and *Hw*BR (*purple solid line*). The UV-visible spectra were measured in the buffer solution containing 50 mm MES (pH 5.8), 4 m NaCl, 0.02% DDM. *AU*, absorbance units. *B*, overall structure of monomeric *Hw*BR. *Nter*, N terminus; *Cter*, C terminus. *C*, top view of wild-type trimeric structure. *D*, top view of wild-type antiparallel dimeric structure. *E*, three-dimensional structure alignment of BR-like proteins. Superimposition of aR-1 (1UAZ; *green*), aR-2 (1VGO; *yellow*), bR (1C3W; *cyan*), dR3 (4FBZ; *purple*), cR-3 (4L35; *blue*), and qR (4QI1; *pink*) structures is shown.

**FIGURE 3.**
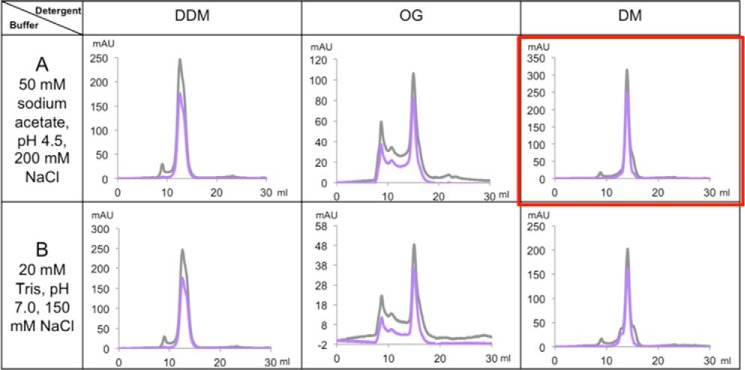
**Buffer and detergent selection of *Hw*BR using size exclusion column.** The *Hw*BR protein was loaded into the size exclusion column (Superdex 200 10/30 GL) and eluted by six combinations of two buffers and three detergents. The *gray line* is the absorption spectra at 280 nm, and the *purple line* is at 552 nm. *AU*, absorbance units.

The *Hw*BR gene was constructed with a C-terminal hexahistidine tag and expressed in *E. coli* C43 (DE3) as described previously (35, 48). The purified *Hw*BR protein in buffer with detergent had a λ_max_ at 552 nm, which is almost consistent with the absorbance value of *Hs*BR monomer, and as a reference, the absorption peak of *Hs*BR trimer, known as purple membrane, showed a peak value at 568 nm, which was red-shifted 13 nm when compared with monomer BR (Fig. 2*A*) (49). Three commonly used detergents, DDM, DM, and OG, in different buffer conditions under pH 4.5 and 7.0, were tested for monodispersity properties of *Hw*BR. The *Hw*BR protein showed two very close peaks in both buffers with 0.02% DDM. In buffers with 0.2% OG, the *Hw*BR protein formed broad peaks. In our detergent screening experiment, *Hw*BR protein showed monodispersed peaks in both buffers with 0.15% DM. After the protein in OG was dialyzed against buffer A with 0.15% DM and loaded onto the column, the elution pattern was restored from the broad peaks to a monodispersed peak in DM (Fig. 3). Therefore, we selected *Hw*BR protein in Buffer A containing 0.15% DM as the sample for crystallization, which yielded purple crystals.

##### Overall Structures and Proton Translocation Path of HwBR

Previously, we crystallized the protein using the vapor diffusion method, and the crystals diffracted to around 7 Å (48). In this work, *Hw*BR proteins were crystallized using the *in meso* method (50), and the proteins packed into parallel trimeric and antiparallel dimeric crystals diffracted to 1.85 and 2.58 Å, respectively (Table 1). The structures of the monomers were almost identical and consisted of seven transmembrane α-helices, two β-strands in the BC loops, and a prosthetic group all-*trans* retinal bound to Lys^224^ via a Schiff base (Fig. 2*B*). The values of root mean square deviation (r.m.s.d.) between monomers of the antiparallel dimer and of the trimer are ∼0.36 Å. The trimeric structure showed that lipids (1-monooleoyl-rac-glycerol, MPG) surrounded each monomer to induce the formation of a self-assembled trimeric structure (Fig. 2*C*). In previous studies, it was shown that lipids control the trimeric structure, conformational flexibility, and photocycle activity of BR (51).

**TABLE 1:** **Data collection and refinement statistics for *Hw*BRs**

	WT *Hw*BR	WT *Hw*BR
**Data collection statistics**		
Beamline	BL15A1, NSRRC	BL44XU, Spring-8
Wavelength (Å)	1.000	1.000
Space group	C 2	C 2
Cell dimensions		
a, b, c (Å)	106.23, 61.26, 119.19	131.94, 29.80, 124.97
α, β, γ (°)	90.00, 116.01, 90.00	90.00, 118.77, 90.00
Resolution (Å)	30.0–1.85 (1.88–1.85)*^a^*	30.0–2.58 (2.67–2.58)
*R*_merge_ (%)	6.3 (52.4)	16.8 (79.1)
*I*/σ*I*	17.7 (2.41)	8.2 (2.0)
Completeness (%)	83.7 (90.1)	97.8 (98.0)
Redundancy	2.9 (3.2)	3.5 (3.1)

**Refinement statistics**		
Resolution (Å)	26.6–1.85	28.87–2.58
No. of reflections	48,537	13,107
*R*_work_/*R*_free_, (%)	19.53/22.80	20.71/22.94
Average B-factors (Å^2^) (No. of atoms)		
Protein	17.3 (6,354)	50.4 (3,487)
Ligand	14.8 (60) (retinal)	38.1 (40) (retinal)
	39.4 (216) (MPG)	57.0 (24) (MPG)
	42.1 (6) (glycerol)	52.1 (8) (ACT)*^b^*
Water	38.8 (743)	57.1 (102)
r.m.s.d. bonds (Å)	0.018	0.015
r.m.s.d. angles (°)	1.78	1.62
Ramachandran statistics (%)		
Favored	99.7	96.9
Allowed	0.3	2.9
Disallowed	0	0.2

**PDB**	4QI1	4QID

*^a^* Highest resolution shell is shown in parentheses.

*^b^* ACT, acetate.

We used 30% glycerol as the cryoprotectant when harvesting the crystals. The presence of glycerol improved the resolution from 4 to 1.85 Å. In the structure, one glycerol molecule was found to be bound with chain B (Fig. 2*C*). The antiparallel dimeric structure had one MPG and two acetate molecules bound around the dimeric protein. A top view of the dimer shows that helices A and B from both monomers form a four-helix bundle for antiparallel dimer formation (Fig. 2*D*). In the overall structures, the BC and FG loops (Fig. 2*E*) have some variations that might control the differences between the superfamilies of BR-like proteins (r.m.s.d. ∼0.3–0.4 Å).

**FIGURE 4.**
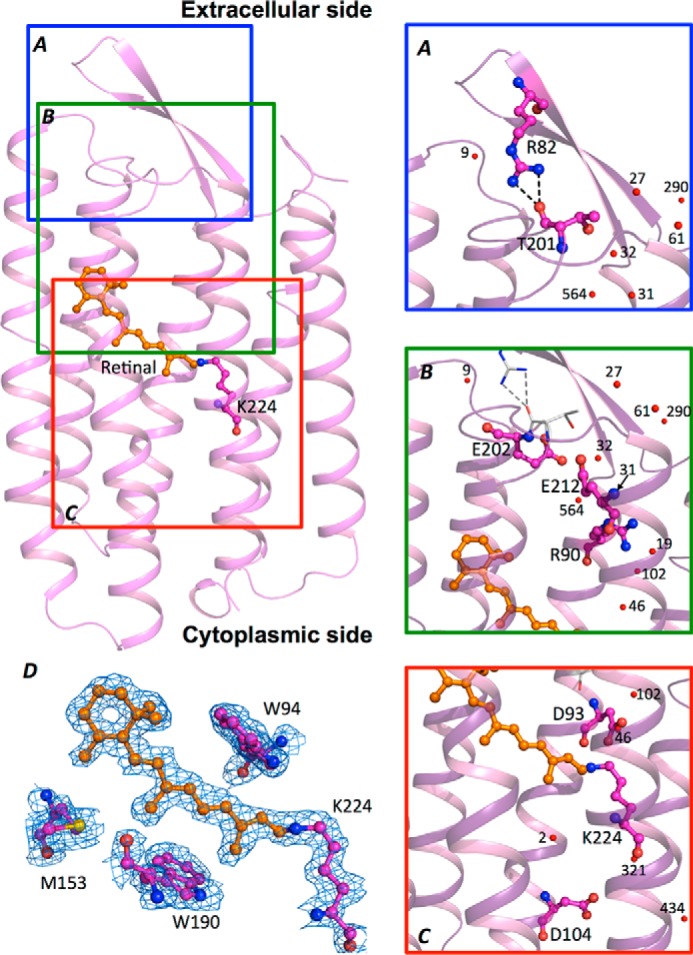
**The structure and proton translocation path of *Hw*BR.** *A*, the proton outward cap region is drawn in a *blue box*, and residues Arg^82^ and Thr^201^ are shown as *sticks*. The hydrogen bonds are represented by *black dashed lines. B*, the residues involved in the proton-releasing group are represented by *sticks* in a *green box. C*, the retinal binding pocket and proton re-uptake residue Asp^93^ are shown in a *red box*. The proton-pumping flow is directed from the cytoplasmic site through the Schiff base to the proton-releasing complex, with protons exiting from the proton outward cap. The waters are shown as *red sphere. A–C*, have enlarged view on the right site labeled with key residues and waters. *D*, electron density maps of retinal and the surrounding region. The 2*F_o_* − *F_c_* electron density map contoured at 1 σ is shown in *blue*. The all-*trans* retinal is shown in *orange stick*, and the key residues surrounded the binding pocket are shown in *magenta stick*.

**FIGURE 5.**
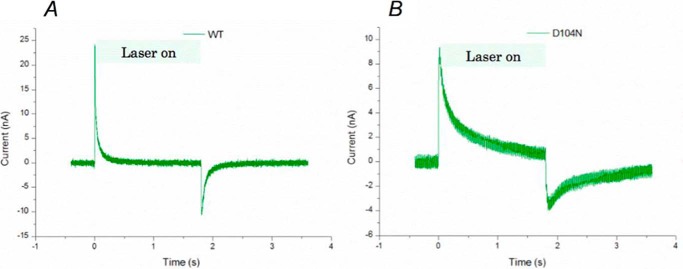
**Light-driven proton translocation activity assay using photocurrent measurements.** *A* and *B*, an indium tin oxide-based photocurrent device was adopted to measure the light-driven photocurrent generation in both wild-type *Hw*BR (*A*) and D104N/*Hw*BR (D96N/*Hs*BR-corresponding mutant) (*B*) at pH 5.8 with 0.1% DDM. A continuous 532-nm green laser was turned on at 0 s and turned off at 1.85 s while the photocurrent was continuously recorded. The *light green shading* indicates the light-on duration. The recovery of photocurrent traces started at time 1.85 s represented the proton reuptake step during the light-driven proton pumping. The recovery half-time (*t*_½_) values of the wild type and D104N/*Hw*BR were around 0.05 and 0.75 s, respectively.

The proton translocation path could be divided into three areas (Fig. 4). On the cytoplasmic side, Asp^104^ (Fig. 4*C*) is the proton uptake accelerator, as seen for all other BR proteins, known as Asp^96^ in *Hs*BR. The D104N/*Hw*BR mutant constructed in this work showed retarded proton uptake during the light-driven proton pumping cycle when compared with the wild-type *Hw*BR (Fig. 5). In the photocycle process, once the retinal binding site was fully protonated, a proton was translocated from Asp^93^ to the proton releasing group, which is composed of Arg^90^, Glu^202^, and Glu^212^, through the hydrogen bond rearrangement matching those observed in other BRs (Fig. 4*B*) (11, 18). In our 1.85 Å resolution structure, all structural waters for the proton translocation path are conserved in *Hw*BR when compared with the 1.55 Å *Hs*BR (1C3W). The proton outward cap (BC loop) at the extracellular site composed of a β hairpin motif was partially sealed by a pair of amide-carbonyl hydrogen bonds formed between Arg^82^ and Thr^201^ (Fig. 4*A*).

In both structures of *Hw*BR solved in this study, two guanidinium nitrogen atoms of Arg^82^ located at the BC loop formed hydrogen bonds with the carboxyl group of the main chain of Thr^201^ in the FG loop in the extracellular region. This β hairpin of the BC loop forms a cap covering the proton translocation channel exit site (Fig. 4*A*). These hydrogen bond connections at this position have never been observed in any other known BR protein (Fig. 1*A*). In our *Hw*BR structures from different crystal packing forms, Arg^82^ was located at the center of the β hairpin in both structures, forming hydrogen bond connections with the main chain atoms of Thr^201^ at the C terminus of helix F.

**FIGURE 6.**
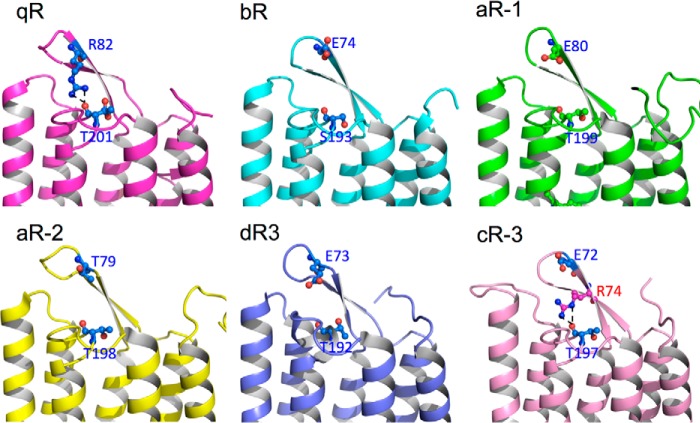
**Comparison of proton outward caps.** The proton outward caps of structures from five BR proteins are presented for qR (*magenta*), bR (*cyan*), aR-1 (*green*), aR-2 (*yellow*), dR3 (*purple*), and cR-3 (*pink*). The residues related to Arg^82^ and Thr^201^ of *Hw*BR are shown as *blue sticks*.

To further investigate the structural residue corresponding to Arg^82^ in other BR proteins, all resolved BR structures were aligned (Fig. 4*E*). Alignment of the BC loop region in *Hw*BR with other structures of aR-1, aR-2, bR, cR-3, and dR3 (23, 25–27) showed that the residues corresponding to Arg^82^ are glutamic acid in all other BR proteins, except in the aR-2, where threonine is the corresponding residue, and no hydrogen bond formation in the corresponding position was observed (Fig. 6). However, there is a hydrogen bond network formed between Arg^74^–Thr^197^ in cR-3 structure. One of the hydrogen bonds in cR-3 linked to the hydrogen atom of the ϵ nitrogen, but two hydrogen bonds in *Hw*BR were formed on the hydrogen atom on η nitrogen. The η nitrogen is sensitive to the micro-chemical environment, and a flexible cap could be formed during proton translocation process.

##### HwBR Is an Optically Stable BR under Wide pH Range and Mutagenesis of Arg^82^ Impairs Optical Stability of HwBR

**FIGURE 7.**
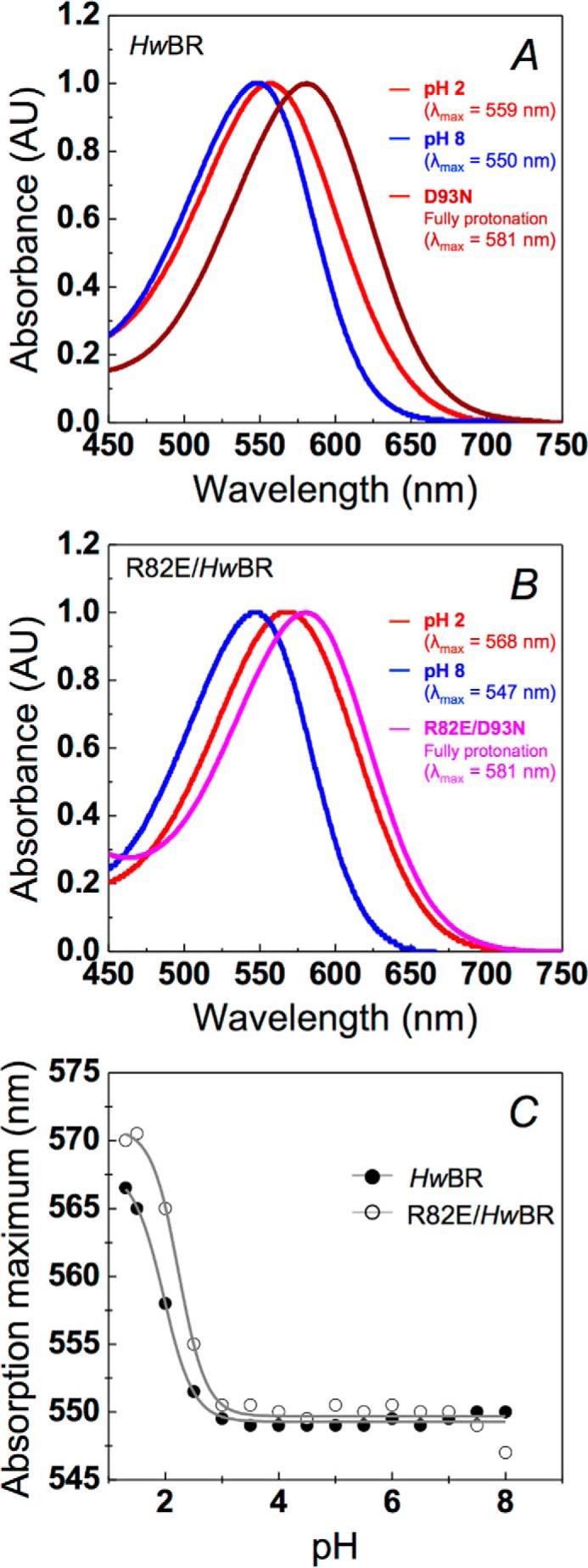
**pH-dependent transitions of wild type and R82E/*Hw*BR in 0.** 1% DDM and 100 mm NaCl. In *A* and *B*, the *red curves* (pH 2) and *blue curves* (pH 8) indicate the spectra of wild type and R82E/*Hw*BR, respectively. The spectra of the putative fully protonated mutant D93N/*Hw*BR (*brown curve*) and R82E/D93N/*Hw*BR (*magenta curve*) are shown in *panels A* and *B*, respectively. *AU*, absorbance units. *C*, pH dependence of absorption maximum of *Hw*BR (*solid circle*) and R82E/*Hw*BR (*open circle*) upon increasing the pH from 1.3 to 8, respectively. Each spectrum was obtained at pH 1.3, 1.5, 2, 2.5, 3, 3.5, 4, 4.5, 5, 5.5, 6, 6.5, 7, 7.5, 8. The pH under 1.3 was inapplicable because of the protein denaturation.

An important feature of rhodopsin is the pH dependence of the maximum absorbance, λ_max_, or activation spectrum, which reflects the micro-environment of the retinal protonation state. Here, we found that *Hw*BR has high optical durability under acidic conditions. *E. coli*-expressed *Hs*BR and *Hw*BR were pre-equilibrated with buffered solution at a pH of 2.0 (Fig. 7*A*, *red*) or 8.0 (Fig. 7*A*, *blue*) for spectral scanning over 250–750 nm. A mere ∼9-nm red-shift in λ_max_ was recorded for *Hw*BR (Fig. 7*A*), significantly less than the ∼55-nm red-shift observed in *Hs*BR (19) under the same conditions. The much smaller red-shift at a pH of 2.0 represents an unusual level of optical stability that has not been observed in BR proteins other than *Hm*BRII (19). To further obtain spectra of the fully protonated counterion states for *Hw*BR, D93N/*Hw*BR, the D85N/*Hs*BR-corresponding mutant proteins, were prepared for comparison (Fig. 7*A*, *brown*). D93N/*Hw*BR showed a red-shifted spectrum with peak at 581 nm similar to that of D85N/*Hs*BR, being red-shifted 20 nm farther than wild-type *Hw*BR in acidic conditions. The result hinted that the micro-environment of the fully protonated retinal-binding pocket was similar between *Hw*BR and *Hs*BR.

To directly corroborate the importance of the Arg^82^–Thr^201^ hydrogen-bonding network in the BC loop of *Hw*BR, a pair of mutants (R82E/*Hw*BR and R82E/D93N/*Hw*BR) was constructed to examine their optical stability under different pH conditions when the hydrogen-bonding network would be disrupted. A 21-nm red-shift was observed for R82E/*Hw*BR, with a λ_max_ value of 568 nm under a pH of 2.0 and a value of 547 nm under a pH of 8.0 (Fig. 7*B*). The fully protonated counterion state of R82E/*Hw*BR was represented by R82E/D93N/*Hw*BR, and its value remained at 581 nm (Fig. 7*B*, *magenta*). To evaluate the spectral shift upon protonation of the Schiff base proton acceptor, titration curves for both *Hw*BR and R82E/*Hw*BR were determined via pH-dependent spectra. *Hw*BR showed a single titration curve with a p*K_a_* of 1.97 (Fig. 7*C*, *solid circle*), which is lower than *Hs*BR and similar to *Hm*BRII (19). Replacement of the arginine with glutamate caused the p*K_a_* to increase to 2.24 (Fig. 7*C*, *open circle*). This result supports our hypothesis that the Arg^82^–Thr^201^ hydrogen-bonding network might have some effect on the retinal Schiff base counterion.

**FIGURE 8.**
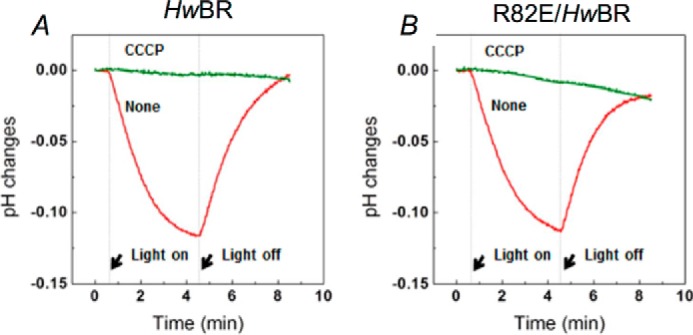
**The light-driven proton pump activity assay of wild-type *Hw*BR and R82E/*Hw*BR.** *A* and *B*, light-driven proton transport by *Hw*BR (*A*) and by R82E/*Hw*BR (*B*) in *E. coli* cells. *Red* and *green lines* indicate the pump activities measured before and after the addition of CCCP, respectively. The *arrow*s show the 1-watt 532-nm continuous green laser stimulation period. The results indicated that the R82E mutant did not interrupt the overall light-driven proton pump activity in *Hw*BR.

A light-driven proton pump activity assay was also conducted on R82E/*Hw*BR to confirm that the mutant does not interfere with the light-driven proton pumping activity. *E. coli* cells transformed with rhodopsins of interest were measured for their light-driven pH change property. The proton pump activity was directly monitored by a pH electrode during light and dark periods. Both wild type and R82E/*Hw*BR showed a pH decrease upon illumination, which was eliminated by the protonophore CCCP, an inhibitor of proton motive force. This result indicated that R82E/*Hw*BR still retains its overall proton translocation ability (Fig. 8).

##### R82E Mutation Slightly Changes the pH-dependent Thermal Stability of HwBR

**FIGURE 9.**
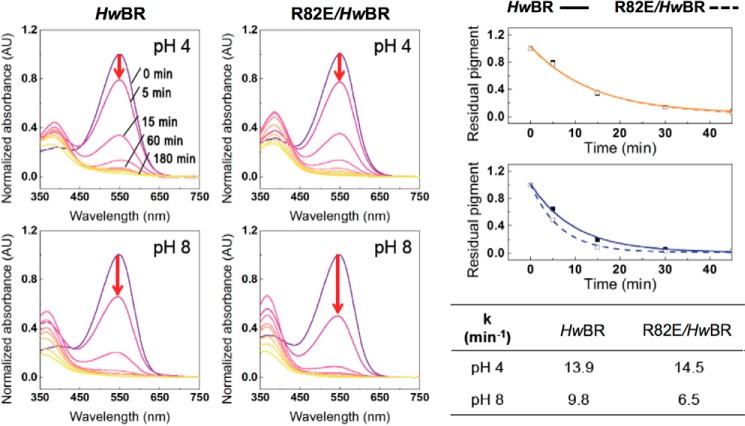
**pH-dependent thermal stability of wild type and R82E/*Hw*BR.** The absorbance of wild type and R82E/*Hw*BR was determined at 0, 5, 15, 30, 45, 60, 90, 120, and 180 min at 75 °C in the buffers at pH 4 and 8, respectively. The time *versus* residual pigment was plotted for wild-type (*solid line*) and R82E (*dashed line*) at pH 4 (*yellow*) and pH 8 (*blue*) to determine the *k* value. *AU*, absorbance units.

The diffusion according to the extracellular proton concentration influences the protonation state of the Schiff base in the ground state. Arg^82^ in *Hw*BR locates on the BC loop and forms a cap above the proton translocation path so that the cap may shield the retinal-binding pocket from outside environment. To investigate this hypothesis, a time-dependent denaturation assay was conducted with slight modifications (43), and the experiments were monitored via spectroscopy at the corresponding λ_max_ of wild type and R82E/*Hw*BR under pH 4 and pH 8 at 75 °C. Both wild type and R82E/*Hw*BR showed a similar time-dependent decrease at pH 4 in 30 min, but R82E/*Hw*BR exhibited a faster denaturation pattern than wild-type *Hw*BR at pH 8 within 15 min (Fig. 9). The results also suggested that the cap together with residue Arg^82^ had a slight effect on the protonation state in the retinal binding pocket.

## Discussion

*H. walsbyi*, a square halophilic archaeon, was first discovered by A. E. Walsby in 1980 (54). Because this unique square morphology halobacterium is abundant in salt lakes around the world, it plays an important role in ecology. Based on the phylogenetic tree from BR protein sequences, new subgroup qR was firstly named as part of *Hw*BR and *Hm*BRII, which were studied by our research team (Fig. 1*B*) (35). Although the overall structure of *Hw*BR is similar to most solved BR structures, a special hydrogen-bonding network located at the extracellular region of the proton pumping path was first found.

**FIGURE 10.**
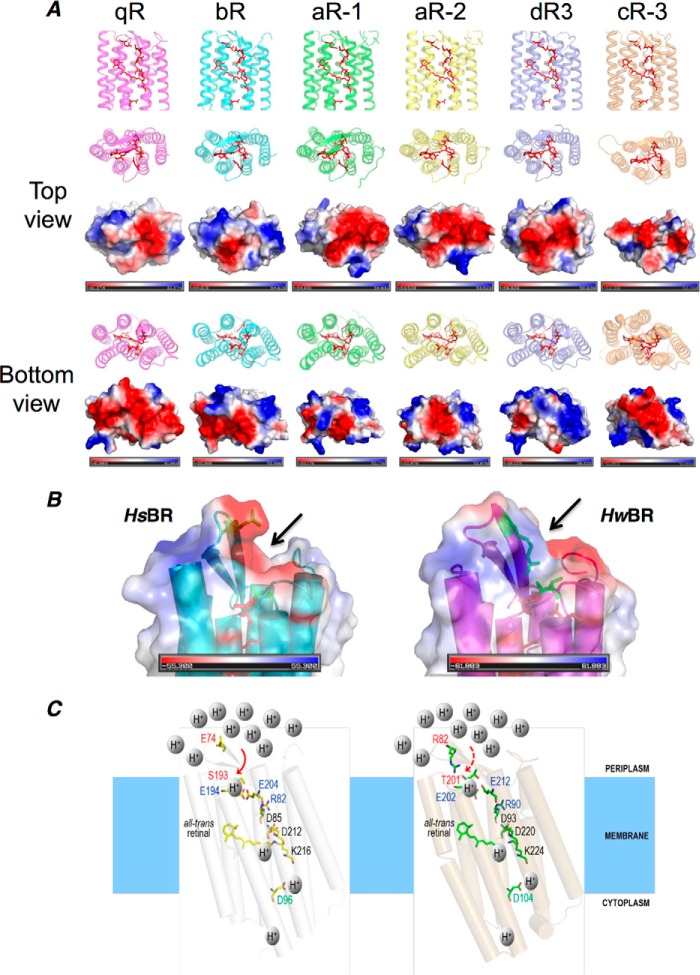
**Electrostatic analysis of different BR structures and the proposed schematic of important proton translocation path residues and BC loop effect of *Hs*BR and *Hw*BR.** *A*, top (extracellular) and bottom (cytoplasmic) views of known BR structures analyzed with respect to their electrostatic charge distribution, using PyMOL. The residues shown in sticks are important during light-driven proton pumping, including the proton-releasing group (Arg^82^, Glu^194^, Glu^204^-corresponding residues), Asp^85^, Asp^212^-corresponding residues, retinal residues, and Asp^96^-corresponding residues numbered in *Hs*BR. *B*, electrostatic analysis of Glu^74^ and Ser^193^ for *Hs*BR and Arg^82^ and Thr^201^ for *Hw*BR. A negatively charged hook region in the BC loop was observed in *Hs*BR but not in *Hw*BR. A flat region in the *Hw*BR BC loop showed a positive charge corresponding to the Arg^82^–Thr^201^ hydrogen-bonding region. *C*, a schematic of *Hs*BR and *Hw*BR with their proton translocation path-related residues under acidic conditions. The *red solid* and *dashed arrows* indicate better or lower accessibility for protons, respectively. Under low pH conditions, the high proton concentration in the extracellular region might access the outlet of proton releasing group (Arg^82^, Glu^194^, Glu^204^) in *Hs*BR (*left*) via diffusion, whereas the Arg^82^–Thr^201^ hydrogen-binding network in *Hw*BR (*right*) can shield the access from the outlet of proton releasing group (Arg^90^, Glu^202^, Glu^212^), thus maintaining the protonation status of the interior and the retinal-binding pocket.

The significance of the Arg^82^–Thr^201^ hydrogen-bonding network within the overall protein surface was summarized by an electrostatic state analysis between *Hw*BR and other BR proteins (Fig. 10*A*). The key Arg^82^–Thr^201^ hydrogen-bonding network sits in the center from the top view. The location of the Arg^82^–Thr^201^ hydrogen-bonding network might shield the retinal-binding pocket from the outside proton equilibrium and protect the protonation condition of both the interior channel and the retinal-binding pocket. In other words, this hydrogen network might prevent the proton acceptor of the Schiff base from outside influences in the ground/resting state, thereby leading to the pH-independent activity spectrum. Worth *et al.* (52) reported that polar and certain charged side chains form hydrogen bonds to main chain atoms in the core of proteins, which is conserved in evolution. For instance, arginine exhibited the highest propensity to form capping interactions that are both conserved and buried at the C termini of α-helices.

When compared with the extracellular side, the cytoplasmic side of *Hw*BR shows a negatively charged region (Fig. 10*A*, *bottom view*, area colored in *red*) with a significantly enlarged surface area among all BRs. Driving the re-uptake of proton from the cytoplasm by the negatively charged region could potentially increase the proton uptake efficiency. Taken together, *Hw*BR has adopted a straightforward approach to achieve a negatively charged region with an enlarged surface area on the cytoplasmic side and a minimized region regulated by the Arg^82^–Thr^201^ hydrogen-bonding network on the extracellular side protecting the retinal-binding pocket micro-environment from the extracellular proton concentration direct influence (Fig. 10, *B* and *C*). Together with these two properties, *Hw*BR seems like a highly efficient machine for proton pumping in acidic condition.

In summary, we characterized the overall important structural and photochemical features in *Hw*BR and in comparison with other known BRs. This study demonstrated how the Arg^82^–Thr^201^ hydrogen-bonding network cap gives *Hw*BR a stable optical property in a wide pH range. The stable optical property might lead to broaden functional pH range of light-driven proton pump activity. This protein property might play an important role in the abundance of *H. walsbyi* cells in salt lakes around the world.

## Author Contributions

M. F. H., H. Y. F., C. S. Y., and A. H. J. W. designed the study and wrote the paper. M. F. H. crystallized and solved the structures. H. Y. F. cloned and purified proteins and performed all activity assays. C. J. C. and H. P. Y. performed light-driven proton pumping activity assay. C. S. Y. and A. H. J. W. supervised the entire project. All authors reviewed the results and approved the final version of the manuscript.

## Footnotes

6 H.-Y. Fu, C.-J. Cai, H.-P. Yi, and C.-S. Yang, unpublished data.

7 Throughout this manuscript, the nomenclatures of aR, bR, cR, dR, and qR are used different bacteriorhodopsin subfamilies, and BR is used for the abbreviation of bacteriorhodopsin protein itself.

## Footnotes

5 The abbreviations used are:

BR: bacteriorhodopsin
*Hw*BR: *Haloquadratum walsbyi* BR
*Hs*BR: *Halobacterium salinarum* BR
*Hm*BRII: *Haloarcula marismortui* BRII
DDM: *n*-dodecyl-β-d-maltoside
DM: *n*-decyl-β-maltoside
OG: *n*-octyl-β-d-glucoside
CCCP: carbonyl cyanide *m*-chlorophenyl hydrazone
MPG: 1-monooleoyl-rac-glycerol
r.m.s.d.: root mean square deviation.
